# Involvement of the IL23/Th17 Pathway in the Pathogenesis of Tunisian Pemphigus Foliaceus

**DOI:** 10.1155/2018/8206983

**Published:** 2018-07-12

**Authors:** M. Ben Jmaa, O. Abida, R. Fakhfakh, E. Bahloul, Kh. Sellami, L. Gaddour, N. Elloumi, M. Ben Ayed, A. Masmoudi, M. Dhouib, M. Abdelmoula, N. Mahfoudh, H. Makni, H. Turki, H. Masmoudi

**Affiliations:** ^1^Immunology Department, Habib Bourguiba Hospital, University of Sfax, Sfax, Tunisia; ^2^Dermatology Department, Hedi Chaker Hospital, University of Sfax, Sfax, Tunisia; ^3^Immunology Department, Hedi Chaker Hospital, University of Sfax, Sfax, Tunisia; ^4^Maxillofacial Surgery Department, Habib Bourguiba Hospital, Sfax, Tunisia

## Abstract

Pemphigus foliaceus (PF) is a rare autoimmune skin disease caused by anti-Dsg1 pathogenic autoantibodies. It is considered as a Th2-mediated disease. Likewise, Th17 cells were recently described in the pathogenesis of the disease but their role is still unclear. We aimed to unravel the eventual implication of the IL23/Th17 pathway in the development of PF. A case-control study was conducted on 115 PF patients and 201 healthy controls using PCR-RFLP and AS-PCR methods. SNPs in *IL23R*, *RORγt*, *IL17A*, *IL17F*, *IL17AR*, *TNFa*, and *STAT3* genes were genotyped. mRNA expression of *IL23R* and *RORγt* was evaluated using Q-PCR. The frequency of circulating Th17 cells was analyzed by flow cytometry. Genetic associations between *IL23R*>rs11209026, *IL17A*>rs3748067, *IL17F*>rs763780, and *TNFa*>rs1800629 and the susceptibility to PF were reported. Moreover, we revealed a significant increased frequency of circulating CD4^+^IL17^+^ cells as well as higher mRNA levels of ROR*γ*t and IL23R in PBMCs of patients. However, no significant increase of ROR*γ*t and IL23R mRNA expression was observed in lesional skin biopsies. In spite of the little size of specimens, our results provide converging arguments for the contribution of the IL23/Th17 pathway in the pathogenesis of PF.

## 1. Introduction

Pemphigus foliaceus (PF) is an autoimmune skin disease with a complex etiology involving genetic and environmental factors [1]. It is mediated by pathogenic auto-antibodies (−Abs) that bind to desmoglein 1 (Dsg1) and lead to acantholysis and consequent blister formation in the subcorneal layer of the epidermis [2-4**]**. The progression from preclinical phase to clinical active disease is associated with the subclass switching of these auto-Abs from IgG1/IgG2 to IgG4 [5–7]. Therefore, and since Th2 cytokines induce B cells to secrete IgG4, PF was considered as a Th2 disease [8, 9] However, this hypothesis was rapidly contradicted by other researchers who detected Dsg1-responsive Th1 and Th2 cells simultaneously in PF patients' blood [10]. More recently, a significant high frequency of CD4^+^IL17^+^ cells in pemphigus patients' PBMCs was reported, particularly in acute onset stage and active chronic stage [11]. Moreover, in PF patients' skin biopsies, while the ratio of IL17^+^ to CD4^+^cell count was 1.8%, IL17-positive cells were undetectable in healthy controls' skin [12]. These data that are in favor of a possible implication of Th17 cells in the pathogenesis of PF were based only on the Th17 common marker to characterize this lymphocyte subset whose differentiation and function involve several other specific markers such as the lineage-specific transcription factor ROR*γ*t and the IL23 receptor (IL23R).

Indeed, the differentiation of Th17cells from CD4^+^CD161^+^ naïve Th cells requires specific cytokines such as TGF-*β*, IL6, IL1β, IL21, and IL23 [13, 14]. In fact, the synergic action of the different cytokines induces the expression of IL23R, which in turn activates the STAT signaling pathway leading to the expression of the transcription factor ROR*γ*t and then the production of Th17-specific cytokines, mainly IL17A and IL17F [15, 16]. Th17 cells play an important role in the clearance of fungal and extracellular bacterial infections by the recruitment of several cell types such as neutrophils, macrophages, and dendritic cells and by the induction of the production of cellular inflammatory mediators, such as TNF*α* [17–19].

The field of action of Th17 cells is not limited to these infections, and there is increasing evidence for the contribution of these cells in the pathogenesis of various autoimmune diseases (AIDs). In fact, IL23 which is considered as the stabilizing factor for Th17 cell commitment seems to play a key role in the acquisition of the pathogenic character of these cells [20]. IL23 signaling through IL23R can amplify Th17 cell response by inducing proinflammatory cytokines, such as IL1*β*, TNF*α*, and IL6, in innate immune cells and by enhancing the activation of STAT3,which is coordinated with ROR*γ*t to stabilize them and maintain their function [15]. Moreover, it was reported that the pathogenicity of Th17 cells was associated with the over activation of the IL23/IL23R signaling pathway [16, 21]. Thus, the IL23/Th17 axis is becoming a leading interesting pathway in the field of AIDs such as systemic lupus erythematosus [22] and pemphigus vulgaris [23] and is one of the most promising targets for AID therapy.

In light of these data and to unfold the involvement of the IL23/Th17 pathway in the pathogenesis of Tunisian PF, we aimed to unravel the eventual genetic contribution of IL23/Th17 genes' polymorphisms and functional association of Th17 cells through the expression of their specific markers.

## 2. Material and Methods

### 2.1. Subjects

One hundred and fifteen PF patients have been recruited since 2002 at the department of Dermatology in Hedi Chaker University Hospital of Sfax, Tunisia. The diagnosis of PF was confirmed by clinical presentation, histopathology (acantholysis in the upper epidermis either in the granular layer or immediately below with subcorneal bullous formation), direct immunofluorescence (IgG and C3 deposits most often located on the whole epidermis and less frequently predominant in the upper layers of the epidermis), indirect immunofluorescence (IgG Abs directed against the epithelial cell surface), and ELISA test for circulatory anti-Dsg1 Abs that was positive for all patients [24].Two hundred and one healthy controls (HC), who did not suffer from any autoimmune or inflammatory disease, were also recruited (see Table 1).

The genetic study is a case-control retrospective study enrolled in the whole population described above. The patient group included 8 men and 107 women, with a mean age of 35 years ranging from 18 years to 84 years. All patients originate from the center and south of Tunisia. The control group included 21 men and 180 women, with a mean age of 39 years ranging from 14 years to 73 years. 

Related patients and/or HC were excluded from the genetic study.

The functional study is a perspective one, conducted on subjects from the population described above that were recruited since 2014. Pemphigus disease area index (PDAI) was measured for all retained patients. We enrolled 13 PF patients and 6 HC for mRNA expression analysis on biopsies, 5 PF women patients and 4 women HC for mRNA expression analysis on PBMCs, and 9 women patients in active stage and 5 women HC for flow cytometry analysis.

The patient group was divided into two groups depending on the stage of the disease: active stage and chronic stage. The active stage of PF is defined as de novo development of new blisters on the skin; none of these patients had yet received immunosuppressive therapy. The chronic stage of PF is defined as the expansion and/or the persistence of existing blisters on the skin. Patients in this stage had already received immunosuppressive treatment.

PF patients suffering from other AIDs were excluded. Healthy subjects were selected based on additional criteria; they have not taken any medicines for at least three weeks before the sampling. All HC were negative for anti-Dsg1Abs.

#### 2.1.1. Sample Collection

After obtaining a written informed consent, samples were collected. Our project was approved by the ethical committee of the Habib Bourguiba University Hospital of Sfax (protocol number of the ethical committee, 4/12).

*(1) Skin Biopsy*. 13 biopsies were taken from 10 patients in the active stage of the disease and from 3 patients in the chronic stage. In addition, 6 control biopsies were taken from healthy subjects undergoing surgeries for a trichilemmal cyst or plastic surgery.

*(2) Blood*. A total of 10 ml of blood was taken from patients and HC in sterile endotoxin-free vacutainers with EDTA as anticoagulant for DNA extraction or in heparinized tubes for mRNA expression analysis and cytometry analysis.

### 2.2. SNP Selection

Tag single-nucleotide polymorphisms (tag SNPs) in the IL23/Th17 axis genes were selected according to their susceptibility to other AIDs and to the genotyping data from the CEU available from the International HapMap project. Selection was undertaken using minor allele frequency (greater than 5%) in Caucasian and sub-Saharan populations.

Four candidate SNPs in the *IL23R* gene in chromosome 1: rs1884444, rs7517847, rs11209026 and rs10889677, two SNPs in the *IL17A* gene in chromosome 6: rs3748067 and rs2275913, rs763780 in *IL17F* in chromosome 6, rs4819554 in the *IL17RA* gene in chromosome 22, rs9645406 in *RORγt* within chromosome 1, rs1800629 in the *TNFa* gene within chromosome 6, and rs744166 in the *STAT3* gene within chromosome 17, were selected. All primers and enzymes used in this study are presented in Table 2.

### 2.3. SNP Genotyping

Genomic DNA was extracted from whole blood samples using a standard proteinase K digestion and phenol/chloroform extraction procedure. Genotyping was performed using the PCR-RLFP method for all SNPs except for rs1884444 in the *IL23R* gene and rs9645406 in *RORγt* which were genotyped using the AS-PCR method (see Table 2).

#### 2.3.1. PCR-RFLP

The PCR amplification was carried out in a volume of 25 *μ*l including 1x buffer, 2 mM MgCl_2_, 0.2–0.4 *μ*mol of each primer (Invitrogen®, CA, USA), 0.12 mM dNTP (Invitrogen, CA, USA),1 U Taq polymerase (Invitrogen, CA, USA), and 50 *η*g of DNA template. Enzymatic digestion was performed in a total of 10 *μ*l mixture reaction containing 1x buffer, 0.1x BSA, and 2 U restriction enzyme (Thermo Fisher®, MA, USA). Primers were designed using primer3 software (http://primer3.ut.ee/). Restriction enzymes were selected using the NEBcutter software (http://nc2.neb.com/NEBcutter2/).

#### 2.3.2. AS-PCR

The primers were designed using WASP software (http://bioinfo.biotec.or.th/WASP). Common primer was labeled with 6-FAM fluorescent labels for the rs1884444 and 6-NED fluorescent labels for rs9645406. The mutant primer containing the mutant base was labeled with a poly(A) tail. The PCR amplification was carried out in a volume of 10 *μ*l including 1x buffer, 2.5 pmol of each primer (Invitrogen, CA, USA), 10 mM dNTP (Invitrogen, CA, USA), 1 U AmpliTaq Gold™ DNA Polymerase **(**Applied Biosystems™, CA, USA), and 1 *η*g of DNA template. Amplified products were run on ABI prism DNA sequencer (PerkinElmer®, CT, USA) and the output file was analyzed using GeneScan software analysis.

### 2.4. Quantitative mRNA Expression of IL23R and ROR*γ*t in the Skin and PBMCs by Q-PCR

Peripheral blood mononuclear cells (PBMCs) were isolated by Histopaque-1077 density-gradient method. Total RNA from PBMCs was extracted using One-step RNA reagent (Bio Basic®, Canada) following the manufacturer's instructions and then reversely transcribed using the High-Capacity cDNA Reverse Transcription kit (Applied Biosystems®, CA, USA). The real-time quantitative PCR was performed via the StepOnePlus Real-Time PCR systems (Applied Biosystems, CA, USA). TaqMan analysis was conducted using predesigned and optimized assays from Applied Biosystems: IL23R (ID: Hs 00332759_m1) and ROR*γ*t (ID: Hs 01076122_m1). PCR reaction parameters were as follows: 95°C for 10 min, followed by 40 cycles of 95°C for 15 s, 60°C for 1 min. All measurements were performed in triplicate. For the relative quantification, data were analyzed by the comparative 2^−ΔΔCt^ method and normalized to the average of housekeeping gene GAPDH.

### 2.5. Flow Cytometry Analysis

PBMCs were seeded into 24-well plates at a final concentration of 10^6^ cells/ml, in RPMI1640 complete medium (containing 10% of the fetal bovine serum, 200 g/ml penicillin, and 200 U/ml streptomycin), and were stimulated with 12-myristate 13-acetate (50 ng/ml) and calcium ionomycin (1 *μ*g/ml), in the presence of GolgiStop protein transport inhibitor (BD Biosciences®, CA, USA) at 37°C for four hours in a 5% of CO_2_ incubator. Cells were subsequently stained for surface markers using PerCP-Cy5.5-labeled anti-human CD4 and PE-labeled anti-human IL17 for intracellular cytokine (BD Bioscience, CA, USA). CD4^+^T cells were gated, and the percentages of these cells producing IL17 were calculated. The BD FACS Canto II System (BD Biosciences, USA) and Kaluza Analysis 1.5a software (Beckman Coulter, USA) were used for analysis.

### 2.6. Statistical Analysis

A case-control analysis was performed using SHESIS software (http://analysis.bio-x.cn) for each SNP and haplotype. Hardy–Weinberg equilibrium (HWE) was assessed in controls using a *χ*2 test with one degree of freedom. A threshold *P* < 0.05 was regarded to indicate deviation from HWE. Odds ratios (OR) and 95% confidence intervals (CI) were calculated for each allele using 2 × 2 contingency tables to estimate the magnitude of association. Binary logistic regression was used to make age and gender adjustment. The linkage disequilibrium (LD) coefficients *D*' = *D*/*D*_max_ and *r*^2^ values for the pair of the most common alleles at each site were also estimated, and high values of LD were defined as *r*^2^ > 0.33 and *D*' > 0.7. The significance level of *P* < 0.05 and odds ratios (OR) with 95% confidence intervals (95% CI) was chosen for all sets.

For functional experiments, statistical analyses were carried out using SPSS20.0 software (IBM SPSS® Inc., IL, USA). As the number of samples was less than twenty, the nonparametric Mann–Whitney *U* test was used to analyze flow cytometry results and mRNA expression of IL23R and ROR*γ*t. Spearman correlation rho “*r*” was used to determine the possible correlation between different results.

## 3. Results

### 3.1. Genetic Association of Investigated SNPs with Susceptibility to PF

Genotype frequencies of all SNPs tested in control subjects were consistent with those expected from the Hardy–Weinberg equilibrium (HWE) except for rs1884444 polymorphism in the *IL23R* gene and rs763780 in *IL17F* gene (*P* < 0.001). Minor allele frequency of all the polymorphisms was consistent with that reported in the HapMap database. The genotypic and allelic distributions of the IL23/Th17 gene polymorphisms as well as their associations with the risk to PF are shown in Table 3.

Concerning the *IL23R* gene, the rs11209026 A>G polymorphism seems to be closely associated with the PF's risk. In fact, the homozygous GG genotype was significantly overexpressed in patients (89.7%) compared to HC (76.2%) (*P* = 0.004, OR = 2.37, 95% CI 1.35–5.53), contrary to the AG genotype which was more expressed in controls (*P* = 0.01, OR = 0.42, 95% CI 0.21–0.87). Likewise, the decreased expression of the A allele observed in the patient group compared to HC suggests its protective role against PF (*P* = 0.001, OR = 0.35, 95% CI 0.18–0.69). No association was found either with rs1884444, with rs7517847, or with rs10889677.

For the *IL17A* gene, a strong association of rs3748067 T>C with the occurrence of PF was revealed. The CC homozygous genotype and the C allele seem to increase the risk of the development of the disease (*P* = 1.17*e* − 04, OR = 5.03, 95% CI 2.34–10.78; *P* = 8.29*e* − 06, OR = 4.57, 95% CI 2.23–9.36). In addition, the heterozygous genotype CT appears as a PF protection genotype (*P* = 9.47*e* − 04, OR = 0.23, 95% CI 0.11–0.50). No significant association was observed for rs2275913.

For *IL17RA* gene polymorphism, no statistical significant difference in genotype and allele frequencies was observed between patients and controls.

As for the *IL17F* gene, a significant increase of the rs763780>C allele was observed in the patient group (10.8%) compared to HC (4.9%) (*P* = 0.007, OR = 2.35, 95% CI 1.24–4.42). On the other hand, the TT genotype was significantly less expressed in patients (89.2%) than in HC (95.1%) (*P* = 0.02, OR = 0.44, 95% CI 0.22–0.91), suggesting its protective role.

Regarding the *TNFa* gene, the allelic distribution of rs1800629 A>G in patients' cohort revealed a great significant increase of the A allele (*P* = 4.06*e* − 5, OR = 2.33, 95% CI (1.55–3.52)). In addition, while the homozygous AA and heterozygous AG genotypes are associated with the PF's risk (*P* = 0.02, OR = 2.76, 95% CI 1.12–6.82; *P* = 0.008, OR = 2.05, 95% CI 1.20–3.51), the GG genotype appeared to be a protector genotype (*P* = 0.0002, OR = 0.38, 95% CI 0.22–0.63).

No significant association was found with rs9645406 in the ROR*γ*t gene or with rs744166 in the STAT3 gene.

All significant associations were conserved after age and gender adjustment using the binary logistic regression.

### 3.2. Linkage Disequilibrium (LD), Haplotype, and Combination Analysis

**Figure 1:**
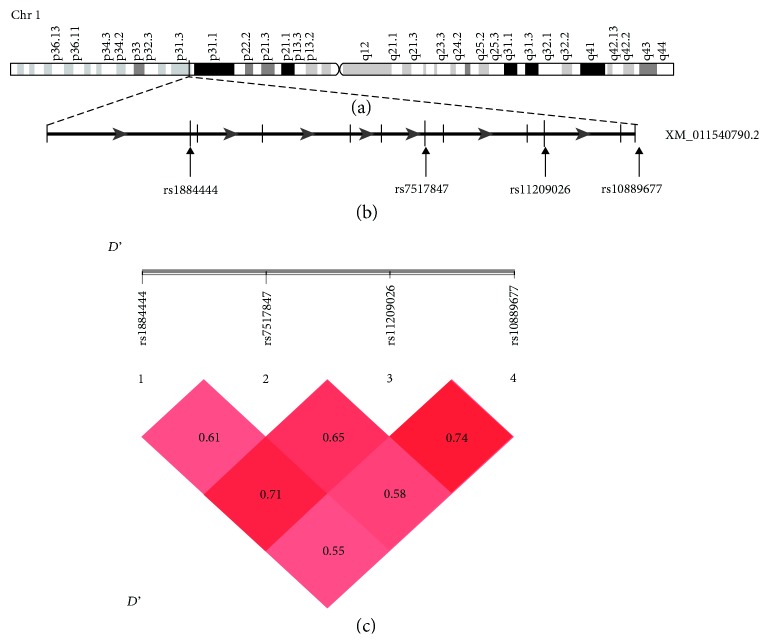
Overview and linkage disequilibrium (LD) on chromosome 1q31.1. (a) Overview of chromosome 1. (b) Schematic structure of the *IL23R* gene. Exons are depicted by black vertical lines. The positions of the four SNPs are shown by the arrows. (c) LD prime charts generated using SHEsis software summarize LD (*D*') patterns between the 4 SNP. *D*' > 0.7 is considered as a high value of LD.

The LD analysis among the patient group was conducted by pairwise comparison of the 4 polymorphisms studied in the *IL23R* gene within chromosome 1 (see Figure 1).

An evidence for LD was revealed between rs11209026 and rs10889677 (*D*' = 0.74) and with rs1884444 (*D*' = 0.71) suggesting that the presumed haplotype of susceptibility to Tunisian PF is rs1884444>T, rs7517847>T, rs11209026>G, rs10889677>A, which contains the rs11209026>G susceptibility allele. This haplotype was more expressed in patients (23.4%) than in controls (16.3%) and, therefore, appears as a risk haplotype to the disease (*P* = 1.71*e* − 04, OR = 2.41, 95% CI 1.51~3.85) (see Table 4).

**Figure 2:**
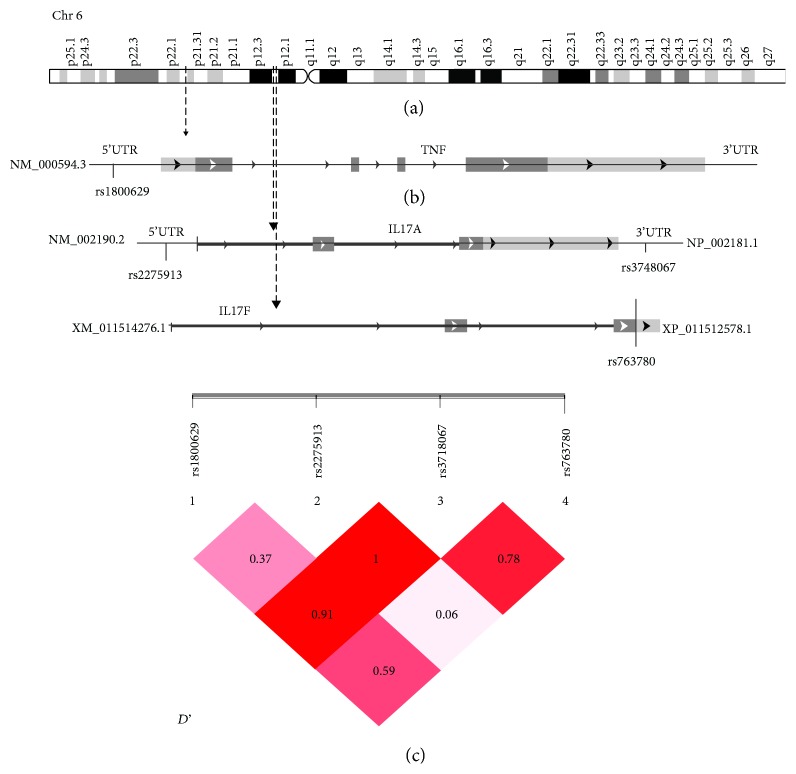
Overview and linkage disequilibrium (LD) on chromosome 6. (a) Overview of chromosome 6. (b) Schematic structure of the *IL17A*, *IL17F*, and *TNFa* genes. The positions of the four SNPs are shown by the arrows (c). LD prime charts generated using SHEsis software summarize LD (*D*') patterns between the 4 SNPs. *D*' > 0.7 is considered as a high value of LD.

Since *IL17A* (rs3748067 and rs2275913), *IL17F* (rs763780), and *TNFa* (rs1800629) genes are located on nearby regions 6p12.2 and 6p21.33, respectively, LD values may explain the possible effect and interaction between their different alleles (see Figure 2).

Thus, an evidence for LD between the SNPs investigated in *IL17A*, *F*, and *TNFa* was observed. Indeed, strong LD between rs3748067 and rs2275913 (*D*' = 1) and rs3748067 and rs1800629 (*D*' = 0.91) were found, indicating that the association observed with these SNPs must be interdependent. Additionally, the estimated frequencies of haplotypes were statistically different between PF patients and controls (global *χ*2 = 23.17, global Pearson's *P* = 1.17*e* − 04). In fact, haplotypes AGCT and GGCT which contain the associated risk allele rs3748067>C (*P* < 0.0001) were associated with an increased risk to PF. In contrast, the GGTT haplotype seems to confer protection against PF (*P* = 0.001) (see Table 4).

On the other hand, possible combinations between the different SNPs were analyzed and a strong evidence for gene-gene interactions between them was revealed (see Table 5). Thus, three associated combinations that differ statically between patients and controls were observed; TTGAAGCTAC (*P* = 0.018, OR = 4.49, 95% CI 1.15–17.57), TGGCGGCTAC (*P* = 0.003, OR = 4.87, 95% CI 1.51–15.74) and TTGAGGCTAT (*P* = 0.017, OR = 0.017, 95% CI 1.16–8.00).

### 3.3. Relative mRNA Expression of IL23R and ROR*γ*t in the Skin and Blood

**Figure 3:**
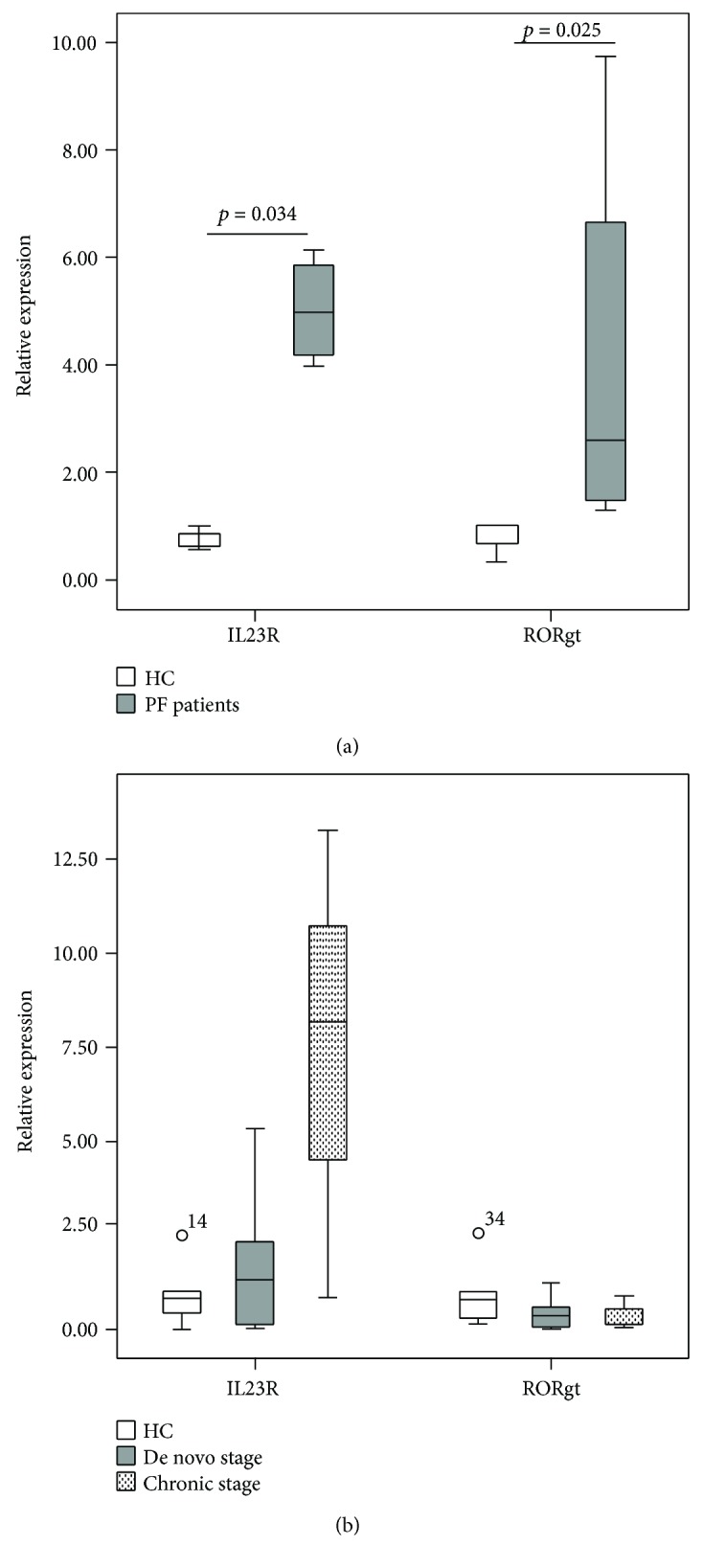
mRNA expression of IL23R and ROR*γ*t in peripheral blood (a) and skin biopsies (b). PBMCs were extracted from 5 de novo patients and 4 HC. Biopsies from de novo patients (*N* = 10), patients in the chronic stage (*N* = 3), and from HC (*N* = 5) were analyzed using Q-PCR. The relative expression was estimated using 2^−ΔΔCt^ method and normalized to the average of the GAPDH housekeeping gene. *P* value was calculated using Mann–Whitney *U* test.

In patients' blood, the relative mRNA expression levels of ROR*γ*t and IL23R were significantly higher (*P* < 0.05) compared to the HC (9.64 ± 12.96 versus 0.778 ± 0.382 and 5.01 ± 1.00 versus 0.75 ± 0.22, resp.) as shown in Figure 3. Moreover, a strong positive correlation was revealed between the expression of these two markers (*r* = 0.943; *P* < 0.005).

In spite of the insignificant difference in ROR*γ*t and IL23R expression levels in skin biopsies between the patient and control groups (1.09 ± 1.74 versus 0.95 ± 0.96 and 2.99 ± 3.89 versus 1.18 ± 0.90, resp.), the IL23R mRNA was slightly more expressed in specimens from patients in the chronic stage than those de novo (7.427 ± 6.25 versus 1.661 ± 1.734) (see Figure 3).

### 3.4. Increased Frequency of CD4^+^IL17^+^ Circulatory Cells in Patients' Blood

**Figure 4:**
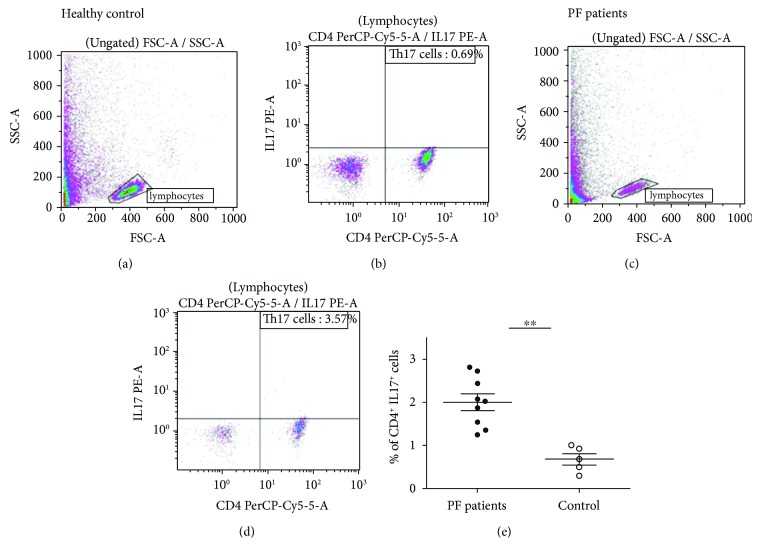
Frequency of IL17^+^ cells in the peripheral blood mononuclear cells of PF patients and healthy controls. Th17 cells identified using specific antibodies CD4-PerCP-Cy5 and IL17A-PE. Representative flow cytometry analysis of Th17 cells in healthy controls (a, b) and in PF patients (c, d). (e) Percentage of Th17 cells in control and patients' PBMCs.

After stimulation, the frequency of CD4^+^IL17^+^cells was significantly higher in patients compared to controls as shown in Figure 4 (*P* = 0.001). In patients, the percentage of Th17 cells varied from 1.36 to 3.57% of CD4^+^cells; however, in the control group, the percentage does not exceed 1% (see Figure 4).

## 4. Discussion

The IL23/Th17 pathway has taken a lot of interest recently, as it is pivotal both in tissue immunosurveillance and autoimmunity mechanisms. Based on genetic and functional studies, strong evidence for its implication in the pathogenesis of several autoimmune diseases, such as systemic lupus erythematosus [22] and rheumatoid arthritis [23], has emerged. Bearing in mind that substitutions of one base pair (SNPs) may have functional importance, we investigated, in the present data, an eventual functional contribution of the most important IL23/Th17 axis markers in the pathogenesis of PF.

Concerning the four Tag SNPs genotyped in the *IL23R* gene, only rs11209026>G was found to be strongly associated with PF susceptibility (*P* = 0.001, OR = 2.81). Moreover, this SNP in strong LD with the others generates the susceptibility haplotype rs1884444>T-rs7517847>T-rs11209026>G-rs10889677>A. Lesional skin biopsies from PF patients showed a slight increase of IL23R mRNA expression as compared to those from HC. Interestingly, specimens of patients in the chronic stage showed a higher mRNA level than those of de novo patients. Furthermore, this relative expression was more significantly pronounced in PF's PBMCs compared to HC ones (*P* = 0.03). The critical role of the IL23/IL23R signaling pathway was proved in favoring terminal differentiation, maintenance, and pathogenicity of Th17 cells [16]. In addition, strong data demonstrate that the lack of the proinflammatory cytokine IL23 in mice makes them resistant to experimental models of arthritis and multiple sclerosis (MS) [25, 26]. Thus, overexpression of this cytokine was reported in several AIDs such as psoriasis [27] and PV [28] but not in PF. The genetic association of rs11209026 was widely described in the context of AID. This variant is located between the transmembrane domain and putative JAK2 binding site in the cytoplasmic portion of IL23R protein and is extremely conserved across different species [29]. Consequently, a change in the highly conserved Arg to Gln might have functional consequences on the IL23R transduction pathway. Several functional data have proved that the protective effect of A allele against autoimmune disease is exerted by the impairment of IL23-induced Th17 cell functions without interfering with Th17 differentiation nor affecting the IL17 production [21, 30]. Unfortunately, as all our patients express the GG genotype, the analysis of the expression in patients with GG genotypes versus others with AG genotypes was not possible.

On the other hand, we found significant associations with genotyped SNPs in the 6p12.2 region in chromosome 6: rs3748067>C in*IL17A* and rs763780>C in *IL17F*. While few studies have explored the association of rs3748067 in *IL17A* with the cancer risk but not with AIDs [31, 32], rs763780 in *IL17F* was associated with many AIDs such rheumatoid arthritis in Tunisian population [33].

Little amount of ROR*γ*t, the specific transcription factor of Th17 cells, was observed in PF patients' skin biopsies, but the difference when compared to HC was not statistically significant. PBMCs from PF patients showed a significantly higher level of ROR*γ*t mRNA, when compared to specimens from HC. A possible explanation for the lack of correlation may reside in the sample in which mRNA was measured and/or in the little size of the skin sample group. Thus, Th17 cells have low-frequency lesional tissue. So, it is possible that ROR*γ*t mRNA is expressed at an undetectable low level in skin biopsies as compared to PBMC. Besides, cellular infiltration in PF includes essentially eosinophils and a little amount of CD4^+^ cells as compared to other autoimmune intraepidermal bullous diseases, such as that in PV [24]. In fact, Arakawa et al. revealed the presence of IL17^+^ cells in PF biopsies with a ratio count to CD4^+^ cell of 1.8%, but the difference compared to HC was not statistically significant. On the contrary, a higher frequency of IL17^+^ cells reaching a level of 5.2% was reported in skin biopsies of PV patients, in which cellular infiltration is more important [12]. As was expected, correlation analysis showed a positive correlation between the expression of ROR*γ*t and IL23R, as the expression of this receptor is necessary for the induction of ROR*γ*t expression. Several data showed that cytokines, including TGF-*β*, IL6, IL1, and IL21, are important in the differentiation of Th17 cells and have proved the necessity of IL23 for the maintenance and the proliferation of established Th17 cells.

For the *TNFa* gene, we found a strong association with rs1800629>A to the PF risk, similar to the result found by Torzecka et al. on Polish population [34]. However, other studies in Indian and Egyptian population estimated no significant genotype and/or allelic frequency difference between PF patients and HC [35, 36]. The investigation of the relation between this polymorphism and secreted levels of TNF*α* showed inconsistent results. Thus, some studies revealed that A allele is associated with a higher level of TNF*α* compared to the common G allele [37, 38], whereas others failed to demonstrate any functional change related to allele change [39, 40]. Previously, we reported the association of an STR within the *TNFa* gene with the PF risk [41]. As the *TNFa* locus is located in the HLA area near the DR and DQ regions, several data suggest that the *TNFa* association with PF could be explained by its linkage disequilibrium with DRB1 locus. However, after multivariant analysis since multiple regression analysis, we showed that, in our population, TNF and HLA DR susceptibility alleles were independently associated with PF [41].

## 5. Conclusion

In this case-control study, a high frequency of circulating Th17 cells was revealed in patients' blood compared to healthy controls. This increased level was confirmed by the mRNA expression analysis of IL23R and ROR*γ*t, specific markers of these cells. Moreover, we reported for the first time, to our knowledge, the genetic contribution of the IL23/Th17 pathway gene's variants in PF pathogenesis. Thus, these results could provide an argument for the contribution of the IL23/Th17 pathway in the pathogenesis of PF. However, much deeper investigation on larger patient groups should be done to support these findings.

## Figures and Tables

**Table 1:** Characteristics of study populations.

Features	PF patients	Controls
Number	115	201
Sex ratio	1/14	1/9
Mean age	35 (18–84)	39 (14–73)
Origin	Center-southern regions of Tunisia	
Anti-Dsg1	Positive	Negative

**Table 2:** Primary information of genotyped SNPs in Th17/IL23 pathway's genes.

Gene SNPs	Base change	MAF	Chromosome regions	Localization	Primers	Enzyme
IL23R rs1884444^a^	G/T nonsens	0.47	Chr1 1p31.3	Exon2	F:Fam^✪^ TGCTCTGTTTCCTTCCTTCC R1:CATCCCATTGAATAGTGACC R2:T_4_CATCCCATTGAATAGTGACA	**—**
IL23R rs7517847	G/T	0.36		Intron6	F:ATTTCTGGATGCCCTTTCCT R:ACATGAATTTGAGGGGCCTA	BbvI
IL23R rs11209026	A/G Arg-gln	0.022		Exon8	F:TTAGACAACAGAGGAGACATTGGA R:CATGTAGTCTAAATCAGAAAACAGAAA	Hpy188I
IL23R rs10889677	A/C	0.39		3′UTR	F:TGCTGGGCCATATGATAAGC R:TCCACCTTCGGGACCTTAAT	MnII
ROR*γ*t rs9645406^a^	T/C		Chr1 1q21.3	Intron	F1:T4CCTCACAGCAAATCTTTTCTC F2:CCTCACAGCAAATCTTTTCTT R:NED^✪^AAAAACCGCTGTAGGGATCA	—
IL17A rs2275913	A/G	0.29	Chr6 6p12.2	5′UTR	F:ATTTCTGCCCTTCCCATTTT R:TTGTGCCTGCTATGAGATGG	EarI
IL17A rs3748067	T/C	0.077		3′UTR	R:GGGGCGAAAATGGTTACGAT F:AAGGCCCCTCAGAGATCAAC	ApoI
IL17F rs763780	C/T	0.093	Chr6 6p12.2	Exon3	F:TGGGTAAGGAGTGGCATTTC R:CTGCATCAATGCTCAAGGAA	NlaIII
IL17RA rs4819554	G/A	0.233	Chr22 22q11.1	5′UTR	F:TGAAATGTGTAATTCGCTGGC R:TGCTTTCCTTGGCTTTGCTT	PvuII
TNF*α* rs1800629	A/G	0.090	Chr6 6p21.33	5′UTR	F:AGGCAATAGGTTTTGAGGGGCAT R:TCCTCCCTGCTCCGATTCC	NcoI
STAT3 rs744166	C/T	0.482	Chr17 17q21.2	Intron	F:GCTGGAGTACAAACCCTGAA R:TGGTATTCAGATGGCGGTCA	AluI

^a^Genotyped using the AS-PCR method.

**Table 3:** Genotype and allele frequencies of IL23/Th17 pathway's genes polymorphisms in Pemphigus foliaceus patients and matched healthy controls.

Gene/SNP	Genotype/allele	Case *N* (%)	Control *N* (%)	*P*	OR	95% CI
IL23R rs11209026	AA	0	5 (2.6)	NS	—	—
	AG	11 (10.3)	41 (21.2)	0.016	0.42	0.21–0.87
	GG	96 (89.7)	147 (76.2)	0.004	2.37	1.35–5.53
	A	11 (5.1)	51 (13.2)	0.001	0.35	0.18–0.69
	G	203 (94.9)	335 (86.8)			

IL23R rs1884444	GG	3 (3)	11 (8.3)	NS	—	—
	GT	80 (79.2)	106 (79.7)	NS	—	—
	TT	18 (17.8)	16 (12)	NS	—	—
	G	86 (42.6)	128 (48.1)	NS	—	—
	T	116 (57.4)	138 (51.9)			

IL23R rs7517847	GG	10 (9.8)	14 (7.4)	NS	—	—
	GT	43 (42.2)	75 (39.7)	NS	—	—
	TT	49 (48)	100 (52.9)	NS	—	—
	G	63 (30.9)	103 (27.2)	NS	—	—
	T	141 (69.1)	276 (72.8)			

IL23R rs10889677	AA	20 (19.8)	40 (22.1)	NS	—	—
	AC	41 (40.6)	71 (39.2)	NS	—	—
	CC	40 (39.6)	70 (38.7)	NS	—	—
	A	81 (40.1)	151 (41.7)	NS	—	—
	C	121 (59.9)	211 (58.3)			

IL17A rs2275913	AA	1 (1)	1 (0.6)	NS	—	—
	AG	9 (9.2)	10 (6.2)	NS	—	—
	GG	88 (89.8)	150 (93.2)	NS	—	—
	A	11 (5.6)	12 (3.7)	NS	—	—
	G	185 (94.4)	310 (96.3)			

IL17A rs3748067	TT	0	4 (3.6)	NS	—	—
	CT	10 (11.1)	38 (35)	9.47*e* − 4	0.23	0.11–0.50
	CC	79 (87.7)	66 (60.8)	1.17*e* − 4	5.03	2.34–10.78
	T	10 (5.5)	46 (21.1)	8.29*e* − 6	0.218	0.10–0.44
	C	170 (94.4)	171 (78.8)			

IL17AR rs4819554	AA	78 (74.3)	137 (71.8)	NS	—	—
	AG	24 (22.9)	48 (25.1)	NS	—	—
	GG	3 (2.9)	6 (3.1)	NS	—	—
	A	180 (85.7)	322 (84.3)	NS	—	—
	G	30 (14.3)	60 (15.7)			

IL17F rs763780	CC	5 (4.7)	3 (1.6)	NS	—	—
	CT	13 (12.3)	13 (6.7)	NS	—	—
	TT	88 (83)	177 (91.7)	0.02	0.44	0.22–0.91
	C	23 (10.8)	19 (4.9)	0.007	2.35	1.24–4.42
	T	189 (89.2)	367 (95.1)			

TNFa rs1800629	AA	12 (12.8)	9 (5)	0.022	2.76	1.12–6.82
	AG	37 (39.4)	43 (24)	0.008	2.05	1.20–3.51
	GG	45 (47.9)	127 (70.9)	0.0002	0.38	0.22–0.63
	A	61 (32.4)	61 (17)	4.06*e* − 5	2.33	1.55–3.52
	G	127 (67.6)	297 (83)			

ROR*γ*t rs9645406	TT	0	0	NS	—	—
	CT	9 (7.9)	13 (6.5)	NS	—	—
	CC	106 (92.1)	188 (93.5)	NS	—	—
	T	11 (4.7)	13 (3.3)	NS	—	—
	C	219 (95.3)	389 (96.7)	NS	—	—

STAT3 rs744166	TT	22 (23.1)	29 (18.1)	NS	—	—
	CT	47 (49.5)	77 (48.1)	NS	—	—
	CC	26 (27.4)	54 (33.8)	NS	—	—
	T	91 (47.9)	135 (42.2)	NS	—	—
	C	99 (52.1)	185 (57.8)	NS	—	—

**Table 4:** Haplotypes of genotyped SNPs on chromosomes 1 and 6.

Chromosome	Haplotype	Case *N* (%)	Control *N* (%)	*P*	OR (95% CI)
1	TTGA	44 (23.4)	39 (16.3)	1.71*e* − 04	2.409 (1.508~3.846)
	TGGC	33 (17.5)	24 (10)	1.16*e* − 04	2.871 (1.648~5.001)
6	AGGT	42 (0.291)	30(0.168)	7.67*e* − 06	3.027 (1.832~5.003)
	GGAT	6 (0.041)	38 (0.213)	0.002	0.276 (0.114~0.664)

**Table 5:** Combinations of genotyped SNPs in *IL23R*, *IL17A*, *IL17F*, *TNFa*, *RORγt*, and *STAT3* showing significant differences between PF patients and controls.

Combinations	Case *N* (%)	Control *N* (%)	*P*	OR (95% CI)
TTGAAGCTAC	7 (5.5)	3 (2.3)	0.018	4.497 (1.151~17.574)
TGGCGGCTAC	10 (7.9)	4 (3.1)	0.003	4.877 (1.511~15.742)
TTGAGGCTAT	11 (8.7)	7 (5.4)	0.017	3.057 (1.167~8.006)

## Data Availability

The data used to support the findings of this study are available from the corresponding author upon request.
